# Survivin-targeted immunotherapy drives robust polyfunctional T cell generation and differentiation in advanced ovarian cancer patients

**DOI:** 10.1080/2162402X.2015.1026529

**Published:** 2015-05-07

**Authors:** Neil L Berinstein, Mohan Karkada, Amit M Oza, Kunle Odunsi, Jeannine A Villella, John J Nemunaitis, Michael A Morse, Tanja Pejovic, James Bentley, Marc Buyse, Rita Nigam, Genevieve M Weir, Lisa D MacDonald, Tara Quinton, Rajkannan Rajagopalan, Kendall Sharp, Andrea Penwell, Leeladhar Sammatur, Tomasz Burzykowski, Marianne M Stanford, Marc Mansour

**Affiliations:** 1 Sunnybrook Health Sciences Centre; Toronto, Ontario, Canada; 2 Immunovaccine, Inc.; Halifax, Nova Scotia, Canada; 3 Princess Margaret Cancer Center; University Health Network; Toronto, Ontario, Canada; 4 Roswell Park Cancer Institute; Buffalo, NY USA; 5 Winthrop-University Hospital; Mineola, NY USA; 6 Mary Crowley Cancer Research Center; Dallas, TX USA; 7 Duke University Medical Center; Durham, NC USA; 8 Oregon Health & Science University; Portland, OR USA; 9 QEII Health Sciences Center; Halifax, Nova Scotia, Canada; 10 International Drug Development Institute (IDDI); Louvain la Neuve, Belgium

**Keywords:** cancer, DepoVax, immunotherapy, survivin, T cells

## Abstract

DepoVax™ is an innovative and strongly immunogenic vaccine platform. Survivin is highly expressed in many tumor types and has reported prognostic value. To generate tumor-specific immune response, a novel cancer vaccine was formulated in DepoVax platform (DPX-Survivac) using survivin HLA class I peptides. Safety and immune potency of DPX-Survivac was tested in combination with immune-modulator metronomic cyclophosphamide in ovarian cancer patients. All the patients receiving the therapy produced antigen-specific immune responses; higher dose vaccine and cyclophosphamide treatment generating significantly higher magnitude responses. Strong T cell responses were associated with differentiation of naïve T cells into central/effector memory (CM/EM) and late differentiated (LD) polyfunctional antigen-specific CD4^+^ and CD8^+^ T cells. This approach enabled rapid *de novo* activation/expansion of vaccine antigen-specific CD8^+^ T cells and provided a strong rationale for further testing to determine clinical benefits associated with this immune activation. These data represent vaccine-induced T cell activation in a clinical setting to a self-tumor antigen previously described only in animal models.

## Abbreviations

CM: central memory
DPX: DepoVax
ELISPOT: Enzyme linked immunosorbent assay
EM: effector memory
HLA: human leukocyte antigen
LD: late differentiated
mCPA: metronomic cyclophosphamide
MDSC: myeloid derived suppressor cell
PBMC: peripheral blood mononuclear cell
SD: study day
TLR: Toll-like receptor
Treg: regulatory T cell

## Introduction

Generating antigen-specific immune and clinical responses to self-tumor associated antigens has been difficult to date.^1,2^ Effective immune activation could be particularly important in this era because of the potential to augment these even further with various checkpoint inhibitors.^3^ However, for the most part there has been very little success so far and most randomized pivotal clinical trials have failed to meet their clinical endpoints.^1^ There are multiple potential explanations for these failures, and they include both clinical design and vaccine-related factors. For example, selection of patients in minimal residual disease settings where cancer related immune-suppression is less prominent and where the cancer is less likely to progress before effective immune activation has been achieved are important clinical factors to be optimized. Selection of cancers where the antigen being targeted is homogeneously expressed and even selected for in the cancer during progression may also influence the success of the approach.^4^ Selection of tumors that have a favorable microenvironment and are likely to be immune responsive is another important consideration. Modulation of systemic immunity and the tumor environment is likely to be a key factor in the success of vaccine approaches.^5^

The immune profile of the vaccine is also a critical factor that influences the ultimate success of the treatment. The vaccine must be able to appropriately stimulate naïve T cells or T cells that are previously anergized to recognize the antigen in order to undergo differentiation to memory cells, and eventually EM and LD T cells that have fully acquired functional capabilities.^6^ This activation process has been well described in pre-clinical models,^7–9^ but not fully described in cancer patients vaccinated with self-epitopes to establish a fundamental mechanism of action for the therapy in patients. To have the potential to mediate clinical antitumor effects, this T cell differentiation of both CD4^+^ T helper and CD8^+^ cytotoxic T cells must be set in motion.^10,11^ The efficacy with which a cancer vaccine is able to achieve this and the robustness and breadth of the responses produced are likely very important pre-requisites for potential clinical success.^12^

The immunogenicity of cancer vaccines may be further enhanced by strategies to optimize the tumor microenvironment and limit patient immune dysfunction.^13^ Strategies to reduce populations of suppressive cells, such as regulatory T cells (Tregs) or myeloid-derived suppressive cells (MDSCs), or to reduce overall T cell anergy or exhaustion are examples of strategies currently under evaluation.^14,15^ Of note, in pre-clinical testing of vaccines in an experimental cancer model, vaccine formulated in the DepoVax-platform has shown promise in selective expansion of antitumor CD8 T cells, but not the cells involved in immune suppression.^16,20^ Given the challenges, combination treatment approaches with a vaccine and immune modulation is both logical and promising.

We have constructed an immune therapy regimen that includes DPX-Survivac, a unique depot-based vaccine formulation containing multiple CD8 epitopes derived from survivin, a well-established tumor antigen.^17,18^ The multiple epitopes have broad HLA applicability and the distinctive formulation facilitates durable responses. In addition to specific antigens, the vaccine uses an optimized water-free oil-based formulation that is designed to activate the innate immune response through a polynucleotide toll-like receptor (TLR) agonist and stimulate the adaptive immune system through a non-specific T helper epitope, in addition to ensuring long exposure of antigen to the adaptive component of the immune system.^19,20^ Importantly, recognizing the importance of immune modulation, we have combined vaccination with metronomic administration of cyclophosphamide, which has been previously shown to inhibit suppressive immune cell populations such as Tregs and boost antigen-specific immune responses induced by vaccination.^21,22^ To optimize clinical factors, we have evaluated this combination approach in patients with ovarian cancer who are in minimal residual disease states post standard of care therapies. The safety and immunologic activation of this approach is described.

## Results

### Demographics and safety analysis

A Phase 1 clinical trial to evaluate the safety and immunogenicity of DPX-Survivac in patients with ovarian cancer either in first or second remission was performed. Subjects received three subcutaneous injections of the vaccine 3 weeks apart in the same upper thigh region. The three cohorts including doses of vaccine/cyclophosphamide and schedules are shown in **Supplemental Figure 5**. The demographics and baseline characteristics of the patients are shown in **Table 1**. The median time from diagnosis was 28 months (range 5–81 months). No major differences in demographic or prognostic factors between cohorts were noted.

**Table 1.** Patient demographics and baseline characteristics for subjects receiving DPX-Survivac with or without cyclophosphamide*

	Cohort A	Cohort B	Cohort C	Total
Number of Subjects	7**	6	6	19
Median Age (Range)	58 (41–72)	54 (35–65)	60 (47–69)	59 (35–72)
Cancer Type: Ovarian	7	6	6	19
Fallopian Tube	0	0	0	0
Peritoneal	0	0	0	0
Stage at Diagnosis: I	0	0	1	1
II	0	1	1	2
III	6	5	3	14
IV	1	0	1	2
ECOG Status: 0	5	6	6	17
1	2	0	0	2
1st Line Patients	5	4	2	11
Recurrent Patients	2	2	4	8
Route of Chemotherapy: IV	4	3	4	11
IP	3	3	2	8
Neoadjuvant Treatment	1	2	1	4
Avastin	0	1	2	3
Measurable Disease	2	2	2	6
Median Time from Diagnosis to Study Day –8 (Range)	13 months (5–53)	15 months (6–81)	39 months (28–46)	28 months (5–81)
Elevated CA-125 (>30 U)	0	2	0	2

* all subjects identified race as “white”; **only 6 subject received a full course

Systemic adverse events were limited to grade 1 severity. The most common systemic adverse event was grade 1 fatigue. Other grade 1 events that occurred in the occasional patient included decreases in white blood cell numbers, chills, lymphadenopathy, arthralgia, decreased mobility, neuropathy, muscle spasm, pyrexia, myalgia, influenza-like illness, and chest discomfort. The most significant adverse events were injection site reactions, with all patients having some type of reaction during the study shown in **Table 2** and **Supplemental Table 1**. There were no significant differences in the injection site induration or erythema between the three cohorts in this study. There were also five patients with injection site ulcerations, which all occurred after the third vaccination. These were either grade 2 or 3. The three grade 3 injection site ulcerations occurred in cohorts B and C, where cyclophosphamide was used. All injection site ulcerations were transient and resolved slowly with residual skin discoloration.

**Table 2.** Most significant injection site reactions: grade after 1, 2, or 3 vaccinations

Cohort	Subject	Induration Highest Grade Following:			Erythema Highest Grade Following:			Ulceration Highest Grade Following:		
		1 Dose	2 Doses	3 Doses	1 Dose	2 Doses	3 Doses	1 Dose	2 Doses	3 Doses
A	02–01	1	2	2	–	1	1	–	–	–
	01–02	–	–	1	–	–	–	–	–	–
	02–03	2	2	2	2	2	2	–	–	2
	09–13	–	–	1	1	1	1	–	–	–
	09–14^*^	2	n/a	n/a	1	n/a	n/a	–	n/a	n/a
	02–18	1	1	1	1	1	1	–	–	–
	01–19	–	1	1	–	1	1	–	–	–
B	02–04	–	–	2	–	–	1	–	–	–
	09–05	–	–	1	2	2	2	–	–	–
	03–06	–	1	1	–	1	1	–	–	–
	03–07	1	1	1	1	1	1	–	–	3
	02–11	–	1	1	1	1	1	–	–	–
	01–12	–	–	–	–	–	1	–	–	–
C	09–08	1	1	2	2	2	3	–	–	3
	10–09	1	1	1	1	1	1	–	–	2
	11–10	1	1	1	1	1	1	–	–	3
	01–15	–	–	1	–	–	1	–	–	–
	02–16	–	1	1	–	2	2	–	–	–
	11–17	1	1	1	1	1	1	–	–	–

* withdrew consent after 1 dose

Eighteen of 19 patients completed full treatment. One patient withdrew consent after a single injection because of local pain near the initial injection site. Patients were consecutively enrolled in the three different cohorts after evidence that each cohort had an acceptable safety profile with less than two dose limiting toxicities per six patients. There were no dose limiting toxicities as defined in the protocol. In cohort C, one patient had a grade 3 injection site ulceration that was reported as a related serious adverse event but as indicated in the Investigator Brochure and protocol, this is an expected adverse event. Another patient required outpatient surgery to debride grade 3 injection site ulceration at 16 weeks post third injection. The debrided tissue at the edge of the ulcer showed an infiltration containing both CD4^+^ and CD8^+^ T cells (**Fig. S1**).

### Immune response

**Figure 1.**
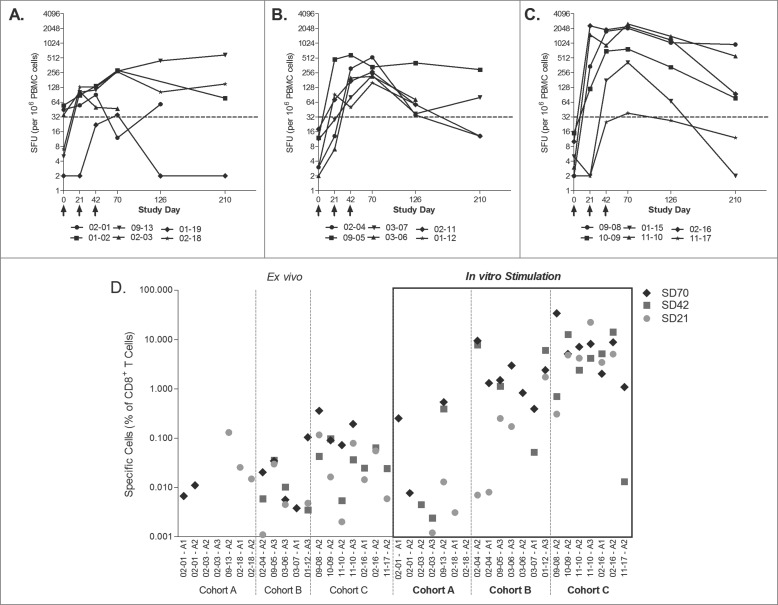
Cyclophosphamide and the dose of vaccine affect the strength of immune response as seen by IFNγ ELISPOT and tetramer staining. PBMCs from cohorts A (**A**), B (**B**) and C (**C**) were stimulated overnight with survivin peptides in an IFNγ ELISPOT assay. Data presented represent the number of spot forming units (SFU) per million PBMC from individual patients over time. Statistically significant differences were established by general linear model: C vs. A, *p* = 0.015; C vs. B, *p* = 0.013. (**D**) Patient PBMCs were stained with corresponding HLA-matched tetramer reagent *ex vivo* (left panels) or stimulated with indicated peptides for 10 d and were stained with corresponding tetramer reagent (right panels) to detect CD8^+^ T cells with peptide-specific T cell receptor repertoires. HIV tetramer served as a negative control and CMV-specific tetramer was used on a known CMV-positive donor PBMC as internal positive assay control (data not shown). Data represented as percentage of live gated CD3^+^CD8^+^ cells that were positive for tetramer staining and the baseline value (Study Day 0) is subtracted from each post-vaccination time points for each subject. The data shows the results at different time points for each patient.

Immune responses were measured rigorously using multiple immunologic assays including Enzyme linked immunosorbent assay (ELISPOT), intracellular cytokine assays and MHC-multimer flow cytometry. CD8^+^ T cell survivin-specific immune responses were induced by DPX-Survivac in most patients. **Figure 1** shows interferonγ (IFNγ) ELISPOT results for all patients who completed treatment. The peak survivin-specific immune responses were induced after one or two vaccinations in all but two patients in cohort A, who required three vaccinations to achieve the peak response. Statistical analysis using an assessment of repeated measurements with a general linear method showed that IFNγ responses were statistically significantly higher in cohort C than those seen in cohort A (where metronomic cyclophosphamide was not used; multiple-testing adjusted *p* = 0.015) and cohort B (which used a lower dose of DPX-Survivac; 0.1 mL versus 0.5 mL, combined with cyclophosphamide; multiple-testing adjusted *p* = 0.013).

**Figure 2.**
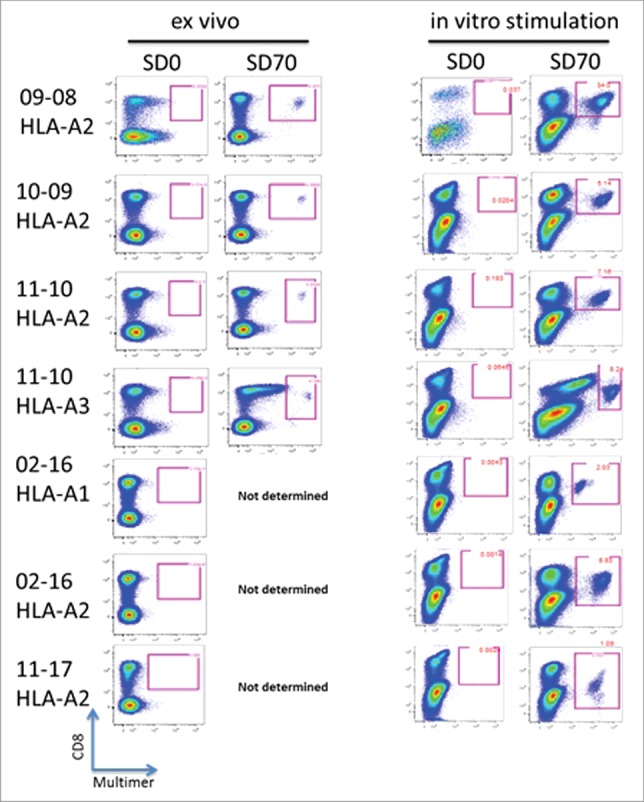
Survivin antigen-specific CD8^+^ T cells are detected in the blood of DPX-Survivac vaccine recipients. PBMCs were tested for the presence of peptide-specific CD8^+^ T cells using MHC-tetramer reagents designed using HLA-A1, -A2 and -A3 survivin peptides used in DPX-Survivac. Assay was performed on non-stimulated PBMC (*ex vivo*) and after 10 d of stimulation *in vitro* in the presence of HLA-matched survivin peptide(s) and low concentrations of IL-2 (10 U/mL) and IL-15 (10 ng/mL). Live lymphocyte gate was used to further identify CD3^+^CD8^+^ T cells that were positive for tetramer staining. Individual patient data from all patients in cohort C are shown. The HLA type of the tetramer used is shown under the patient identification number at the left of the figure.

Tetramers were used in direct *ex vivo* analysis of peripheral blood mononuclear cells (PBMC) from vaccinated patients at different time-points (**Fig. 1D**). CD8^+^ T cell responses were seen in all patients in multiple post vaccination time points. These analyses also showed that the CD8^+^ T cell responses from patients in cohort C appeared greater than those seen in the other two arms. As expected, the PBMCs reacted only with tetramers directed to peptides presented within the appropriate patient MHC and not tetramers to foreign MHC types (data not shown). **Figure 2** shows the flow cytometry assessments for survivin tetramer and CD8 positivity in the patients treated in cohort C. Distinct populations of antigen-specific double positive cells were induced and measurable in the peripheral blood post treatment. These populations were further expanded by a 10 d *in vitro* stimulation with corresponding HLA-matched peptide(s). These populations could be detected both with tetramers using the modified peptide epitope that was used in the DPX-Survivac vaccine as well as the native non-modified survivin peptide (**Fig. S2**).

**Figure 3.**
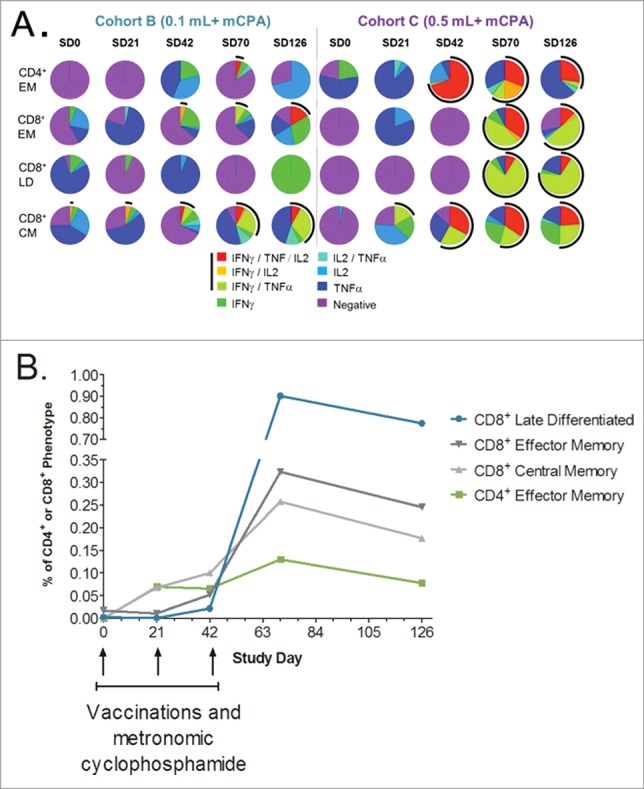
DPX-Survivac vaccination induces polyfunctional T cells and the strength of immune response correlates with Progression Free Survival. (**A**) PBMCs were tested for the presence of multiple cytokine secreting polyfunctional T cells by intracellular staining. After 6 h stimulation with survivin peptides, cells were stained for surface phenotypic markers and intracellular cytokines (IFNγ, TNF-α, IL-2 and others). Flow cytometry analysis was used to detect multiple cytokine production by effector memory (EM; CD27^−^CD45RA^−^), central memory (CM; CD27^+^CD45RA^−^) and late differentiated (LD; CD27^−^CD45RA^+^) CD4^+^ or CD8^+^ T cells at each time point. Each pie chart shows the relative levels of polyfunctional T cells in cohorts B and C at baseline and post-vaccination time points (mean values of all subjects). Arcs indicate the frequency of IFNγ^+^ T cells that can also concurrently secrete one or more additional cytokines (TNF-α/IL-2). (**B**) PBMCs were tested for the presence of polyfunctional T cells of different phenotypes by intracellular cytokine staining. Mean frequency of each phenotype of CD4^+^/CD8^+^ T cells capable of multi-cytokine secretion were measured in cohort C to understand the kinetics of changing functional T cell phenotypes following DPX-Survivac treatment. Data shown represent values after the background staining is subtracted (background on all samples <0.02%).

The functionality of the various antigen-specific CD4^+^ and CD8^+^ T cell phenotypes (CM, EM, LD) was also explored, by intracellular cytokine analysis (**Fig. 3A**). All patients in cohort C produced antigen-specific CD4^+^ and CD8^+^ T cells of the various phenotypes, the majority of which (>50%) were polyfunctional, producing at least two cytokines, one of which being IFNγ. In contrast, in cohort B (with lower vaccine dose), only polyfunctional antigen-specific CM and EM CD8^+^ T cells were detected with this assay. These cells were also detected at a lower percentage than in cohort C (20–30%), and only following the third vaccination. We saw a temporal evolution of polyfunctional CM CD8^+^ T cells initially and the concurrent generation of EM CD4^+^ T cells. Finally, after the third vaccination, CM and EM CD8^+^ T cell were expanded and to a greater extent, LD CD8^+^ polyfunctional T cells were induced (**Fig. 3B**). These were not seen at earlier time-points suggesting a differentiation and rapid expansion of CM CD8^+^ T cells into these two phenotypes (**Fig. S4**). All three CD8^+^ T cell phenotypes persisted for at least 3 months following the third and last immunization. CD4^+^ T cell response to T helper epitope (A16L) was similar in all three cohorts in the study (data not shown) and this epitope served as internal control for vaccine response in these patients.

### Effect of metronomic cyclophosphamide

Despite the addition of metronomic cyclophosphamide in cohorts B and C, there was no significant change in numbers of Treg cells in the blood of treated subjects (**Fig. S3**). However, while there was no noticeable change in the absolute numbers of Tregs in cohort A (without cyclophosphamide), subjects in cohorts B and C showed transient, but not statistically significant, decreases in absolute Tregs during cyclophosphamide treatment (data not shown). The level of absolute Treg cells returned to pre-treatment levels once the cyclophosphamide treatment was stopped. Similarly, we did not see any sustained change in MDSCs. The percentage of B cells did not change in any arm with vaccination. Thus, the mechanism of enhanced CD4^+^ and CD8^+^ T cell activation (shown above) was not related to significant alterations in the numbers of these potentially suppressive immune subsets in the blood. We did not test for the suppressive activity of these subsets of cells. Changes in Tregs and MDSCs were not assessed at tumor sites where it has been shown these cells are most frequent and active.^23,24^

### Clinical activity

As this was a Phase 1 clinical trial, evaluation of clinical activity was not a primary objective. Most patients in this trial did not have measurable disease or CA-125 biomarkers that could be evaluated throughout treatment. Of the five patients with measurable disease or two with elevated CA-125, no patient demonstrated a classically documented objective response or CA-125 reduction. However, at the 6 month follow-up period, 12 of 18 patients remained without clinical progression (i.e. had stable disease). These patients are continuing to be monitored for disease progression.

## Discussion

In this manuscript, we have demonstrated that the novel vaccine formulation DepoVax, containing survivin antigens, together with oral low dose metronomic cyclophosphamide has generated polyfunctional antigen-specific responses in almost all patients treated with this combination. The level of immune activation observed both quantitatively and qualitatively is unprecedented in a cancer population. We have established that this combination treatment is able to generate antigen-specific memory and polyfunctional effector T cells. Antigen-specific immune responses were documented at multiple time points using multiple immune assays. These responses were dose-related and the metronomic cyclophosphamide regimen clearly enhanced the vaccine-induced immune responses. Others have shown that a similar metronomic cyclophosphamide regimen was safe and enhanced immune activation,^21^ but this is the first clinical demonstration of its ability to significantly enhance vaccine-induced T cell responses in the clinic. Others have also shown that antigen-specific immune responses can be generated to survivin using various vaccine formulations^25,26^ but the breadth and robustness of the response was not as strong as in the current approach and the generation of polyfunctional T cells was not addressed.

While metronomic cyclophosphamide has been reported to selectively reduce Tregs,^21^ this effect is not consistently observed in clinical trials.^38^ We did not see any major reduction in Tregs in the peripheral blood, only a transient and non-significant reduction in absolute Tregs during cyclophosphamide treatment. Neither did we see an expansion of Tregs with the DPX-Survivac vaccination. It is possible that changes in Treg number occurred at the tumor site. It is also possible that there was a reduction in the function of Tregs in either the blood or at the tumor site. We did not assess these possibilities. Given the broad biological function modifying activities of cyclophosphamide, it is possible that other undefined mechanisms influencing the effector T cell activation and maturation by the metronomic cyclophosphamide may be involved in the present context.

The detailed flow cytometry and intracellular cytokine data allowed us to document the differentiation process from naïve to LD cytotoxic CD8^+^ T cells post vaccination therapy. There has been some controversy as to whether LD, antigen-specific T cells are the final stage of lymphoid differentiation or whether they can de-differentiate and give rise to antigen-specific CM T cells.^6^ Pre-clinical investigation has established that central and EM T cells have a diminished capacity to proliferate and cannot de-differentiate, however the most effective cytotoxic cells are in this state.^27,28^ The importance of memory CD4^+^ T cells in maintaining memory CD8^+^ T cell responses induced by vaccination has also been recently demonstrated.^10^ Our clinical data demonstrate the sequential development of these memory subsets and supports the notion that antigen-specific LD CD8^+^ T cells are the final stage of the differentiation process and persist supported by memory CD4^+^ and CD8^+^ T cells. Others have also shown that polyfunctional LD T cells are required to mediate clinical activity in the generation of HIV-specific immune responses,^29^ yet the generation of these cells has yet to be described in the setting of cancer. In this study, we detected these polyfunctional cells in all studied patients of cohort C. We postulate that continued presentation of antigen in the DepoVax depot formulation fuels the efficient expansion and sequential differentiation of CM CD8^+^ cells into EM and LD CD8^+^ cells, all of which persisted in the periphery.

A pre-clinical investigation of oil-based emulsion vaccines indicated extensive recruitment and sequestration of antigen-specific CD8^+^ T cells within the vaccine depot.^30^ In these murine studies, extensive trafficking of antigen-specific T cells to the injection site was documented. In addition, this trafficking was so significant that antigen-specific T cells could not be found in the circulation. A clinical investigation of emulsion vaccine site microenvironment also documented a high level of dysfunctional antigen-specific CD8^+^ T cells, although in human subjects responsive antigen-specific CD8^+^ T cells could still be detected in the PBMC.^31^ This prompted the authors to conclude that alternate vaccine formulations need to be investigated for optimal CD8^+^ T cell induction. DepoVax displays distinct kinetics of antigen release compared to water-in-oil vaccines.^32^ A defining feature of DepoVax is that it is a non-emulsion water-free formulation whereby the oil phase retains the antigen, T-helper peptide and adjuvant facilitating prolonged engulfment by antigen-presenting cells, which traffic to the draining lymph node for T cell activation. The persistence of antigen-specific T cells found in PBMC for several months in this study also support the hypothesis that this formulation does not result in complete sequestering of antigen-specific CD8^+^ T cells within the vaccine microenvironment.

We have established that the DPX-Survivac therapeutic vaccine is safe and immunogenic. There were no serious systemic adverse events. Grade 1–2 injection site reactions were most common and in some patients grade 3 injection site reactions occurred. Although grade 3 injection site reactions are not desirable, the biopsy of the injection site with the infiltrate of CD4^+^ and CD8^+^ T cells suggest that these skin ulcerations are “on target” toxicities. These ulcerations occurred at the time of peak survivin-specific immune responses. Ulcers were more severe at the later vaccine sites, which are expected to have more survivin antigen when systemic survivin-specific immune responses were present. We speculate that the injection site reactions in general are a surrogate biomarker of robust immune activation. We are currently exploring other dose levels and scheduling of the vaccination to reduce the frequency of grade 3 injection site ulcerations.

In summary, we have described a highly immunogenic cancer vaccine combination therapy represented by DPX-Survivac and metronomic cyclophosphamide that generates high levels of polyfunctional T cells to self-tumor antigens. This vaccine combination may be an ideal candidate for further combination with other promising immunotherapeutics such as check-point inhibitors.

## Methods

### Patient population and trial design

Patients were included with stage IIc–IV ovarian, fallopian tube or peritoneal cancer with evidence of a complete or partial response by radiological imaging after initial debulking surgery and platinum based cytotoxic therapy or patients with recurrent ovarian, fallopian tubel or peritoneal cancer, who have at least clinically or radiologically stable disease after completion of chemotherapy or surgery for their recurrent disease; HLA-types of enrolled subjects were determined for immune monitoring purposes, but not as an inclusion/exclusion criteria. Subjects who met other standard inclusion/exclusion criteria with a life expectancy of at least 6 months were included in the study. Survivin expression in the cancer was not an inclusion criterion because of the known high frequency of survivin expression in ovarian cancer.^18^

Subjects received three subcutaneous injections of the vaccine 3 weeks apart in the same upper thigh region. The three cohorts including doses of vaccine/cyclophosphamide and schedules are shown in **Supplemental Fig. 5**. If a subject experienced a grade 2 injection site reaction, the vaccine was injected in the alternate upper thigh. The study was conducted in accordance with ethical guidelines of the Declaration of Helsinki. The protocol and patient-informed consent from received approval by individual Institution Review Boards or Research Ethics Boards. Written informed consent was obtained from all patients.

### Vaccine formulation

Survivin-derived peptides and amino acid substitutions of modified peptides are listed elsewhere.^25,26^ The vaccine containing these synthetic peptides (Grindus AG, Torrance, CA and PolyPeptide Laboratories, San Diego, CA) and a T helper peptide epitope^33^ (modified tetanus toxin peptide, 830–844; AQYIKANSKFIGITEL; A16L, PolyPeptide Laboratories) was formulated along with a polynucleotide-based adjuvant in a proprietary DepoVax™ formulation as described in published work.^19,20^ The immunogenic components were encapsulated in a liposomal solution prepared with 0.5 M sodium acetate, pH 9.5, which was then sized (<120 nm) by high pressure extrusion to facilitate sterile filtration of the liposome. The sterile bulk was then aseptically freeze dried in suitable vials and shipped to clinical sites along with a vial of Montanide ISA 51 VG (SEPPIC, France), and the vaccine was stored at 5°C until use. Just before use, the lyophilized vaccine was reconstituted in Montanide ISA 51 VG for injection.

### Immune monitoring

DPX-Survivac-induced immune responses in the peripheral blood of vaccinated patients were investigated at baseline (Study Day (SD) 0, before the first dose) and following each of the additional two doses administered at 3 weeks interval (SD21, SD42) and at SD73, a month after the third and final vaccination. Follow-up samples were also processed at SD126 and SD210 based on sample availability. Peripheral blood from a healthy control female donor was provided by the local blood bank (age 53 years). Whole blood was collected by venipuncture using sodium heparin blood collection tubes at each time point. PBMCs were prepared at the clinical sites by Ficoll-density gradient separation within 4–6 h of collection. PBMCs were suspended in 10% DMSO (Sigma, catalog#D2655)/90% heat-inactivated fetal bovine serum (HI-FBS, VWR, catalog# 95025-550), gradually frozen overnight at −80°C in an isopropyl alcohol bath, then transferred to liquid nitrogen. Cryopreserved PBMCs were transported to ImmuneCarta Services (Montreal, QC) and Cellular Technologies Limited (C.T.L; Shaker Heights, OH) for immunological assessment in the vapor phase of a liquid nitrogen dry shipper (MVE, catalog#10777411). Upon arrival, cells were stored in liquid nitrogen containers until further use (for about 2–3 months). Guidance of lab operations for C.T.L. and ImmuneCarta laboratories are available on the corresponding company websites.

### IFNγ ELISPOT

IFNγ ELISPOT analysis was performed at C.T.L following established SOP. The assay was carried out in serum-free CTL-Test™ media (C.T.L., catalog# CTLT-010, C.T.L) supplemented with 1% L-glutamine (Gibco, catalog# 25030-081). ELISPOT plates (Millipore, catalog# S2EJ011M99) were pre-coated with anti-IFNγ capture antibodies (4 μg/mL, Thermo Scientific, catalog# M700A) diluted in PBS overnight at 4°C, followed by repeated washes. The following stimulators were added to the plate in 100 μL volume per well: CTL-Test media negative control, HLA-matched individual survivin peptides (50 μg/mL and 5 μg/mL), pool of all five survivin peptides (final concentration of each 50 and 5 μg/mL), irrelevant HLA-A2-restricted peptide control peptide (50 μg/mL) or PHA mitogenic positive control (Sigma, 5 μg/mL). PBMCs were thawed and viability assessed using an automated Guava Cell Counter (model PCA-96); viability ranged from 72–98%. Sex-matched healthy control PBMC were included in each assay. PBMC were resuspended at 3×10^6^ cells/mL and added to the ELISPOT plate at 100 μL per well and treated in duplicate. Plates were incubated at 37°C/ 7% CO_2_ for 24 h, then washed rigorously and incubated at room temperature in a humidified box overnight (˜18 h) with biotinylated anti-IFNγ (4 μg/mL, Thermo Scientific, catalog# M701B). Next day, plates were washed rigorously and developed using a streptavidine-HRP complex (BD, catalog# 554066) and AEC substrate (Sigma, catalog# 106380).

Plates were evaluated using the C.T.L. ImmunoSpot® automated reader system (model S5PR). The results of this test were not accepted if the spot counts from the negative control wells were ≥25% of the spot counts in the PHA positive control wells. Artifacts and faint small background spots observed in the negative control wells were excluded. All obtained counts were audited and reviewed. The counting strategy was reviewed by an independent scientist. The assays were conducted in GLP-compliant manner. Mean cut-off for positive response was determined based on Mean ± 2SD values from unstimulated samples from the same subject with at least 2-fold increases from background values. Response definition was not pre-determined due to the possibility of pre-existing immunity; however post-treatment response was compared over pre-treatment response to determine vaccine-induced de-novo response to survivin.

### Tetramer staining

Tetramer analysis was performed by ImmuneCarta Services following established SOP. Custom-made tetramer reagents conjugated to R-phycoerythrin (PE) specific to SurA1.T, SurA2.M and SurA3.K peptides were obtained from TC Metrix SARL (A1, 110882; A2, 110067; A3, 110084 and WT-A2, 110093) and Beckman Coulter (A1, H1203027; A2, H1203034; A3, H1204060). These reagents were validated using patient and control PBMC. Tetramers toward the SurA24 and SurB7 peptides showed some non-specific binding and could not be validated and were not used in this study. PBMC from patients were thawed, counted, viability tested and rested overnight (˜18 h) at 37°C, 5% CO_2_ at a concentration of 10^6^ cells/mL in complete RPMI 1640 media (Gibco, catalog# SH30027FS) containing 10% HI-FBS, 25 mM HEPES (Gibco, catalog# 15630-080), 2 mM L-glutamine (Gibco, catalog# 25030-081), 100 units/mL penicillin/streptomycin (Sigma, catalog# H4034) and 50 mM 2-mercaptoethanol (Sigma, catalog# M7522). Rested PBMC were analyzed for tetramer positive CD8 T cells either directly (*ex vivo*) or after *in vitro* activation for 10 d. For *in vitro* activation, PBMC were resuspended at 1 × 10^6^ cells/mL in complete RPMI media supplemented with 10 IU/mL of IL-2 and 10 ng/mL of IL-15 (both from Peptrotech, IL-2, catalog# 200-01; IL-15, catalog# 200-15). HLA-matched peptide(s) included in the vaccine was used to stimulate cells (10 µg/mL from day 0–3 and 1 µg/mL from day 4–6 with no additional peptide added from day 7–10) in deep well V-bottom plate. Tetramer staining was carried out in PBS (Hyclone, catalog# SH30256.01), supplemented with 2% HI-FBS, 1 mM EDTA (Sigma, catalog #E5134) using overnight rested or 10 d stimulated cells. Between 0.5 × 10^6^ and 1 × 10^6^ cells were used per test in 100 μL buffer. Details of fluorochrome-conjugated antibody suppliers, catalog numbers/clones are listed in **Supplemental Table 2**. Cells were incubated with corresponding tetramer reagent at a concentration recommended by the corresponding supplier for 30 min at 4°C, followed by washing and subsequent staining for surface markers using the antibodies CD3, CD8 and CD45RA in 100 μL staining buffer. Cells were washed again and resuspended in PBS and kept at 4°C until acquisition. For controls, each sample was stained using irrelevant peptide HIV-tetramer. To validate the procedure, known CMV^+^ donor PBMC was stained using CMV-tetramer. Samples were acquired the same day using flow cytometer (LSR II, BD Bioscience) and analyzed using FlowJo software (FlowJo LLC, Ashland, OR). Gating strategy used for flow cytometry is outline in **Supplemental Figure 6**. Assay was validated before patient samples were analyzed and performed under GLP guidelines. Based on negative control tetramer reagent used, a Mean ± 2SD cut-off value of 0.04% was used to determine tetramer positive response.

### Intracellular cytokine staining

Intracellular cytokine staining was performed by ImmuneCarta Services following established SOP. PMBCs were thawed and rested overnight as described for tetramer staining. Next day, cells were stimulated for 1 h with individual peptides and pools of peptides (1 μg/mL) in the presence of anti-CD107a antibody. Experimental controls included unstimulated PBMC, PMA (5 ng/mL, Sigma, catalog # P8139) plus Ionomycin (1 μg/mL, Sigma, catalog # I3909) and CEF (CMV/EBV/FLU, Anaspec, catalog # 61036-05) peptide pool-stimulated PBMC from a known CMV^+^ donor. GolgiStop™ (BD Bioscience, catalog# 554724) was added after 1 h of stimulation, and cells were incubated for an additional 5 h at 37°C and 5% CO_2_. Cells were washed and resuspended in staining buffer (PBS/ 2% FBS/1 mM EDTA). The following surface staining antibodies were added to the cells in 100 µL volume: CD8, CD27, CD3, CD4, CD45RA and a viability marker (Acqa, Invitrogen). After incubation at 4°C for 30 min, cells were washed in staining buffer. Cells were fixed and permeabilized using the commercial kit (BD Bioscience, cytofix/cytoperm, catalog # 554714). Cells were washed twice and resuspended in 100 μL of permeabilization buffer containing intracellular staining antibodies: IFNγ, TNF-α, IL-2, IL-17 and granzyme-B. Cells were incubated for 30 min at room temperature, with vortexing every 10 min, then washed once more, and resuspended in PBS+2% BSA for acquisition. Cells were kept cold and dark until acquisition, which was performed within 24 h. Labeled cells were acquired on a flow cytometer using the FACS DiVa software (BD Bioscience) and the acquired FACS files were analyzed using FlowJo software. Multi-parametric flow analysis was performed after stringent gating of each cytokine positive population and to identify CM, EM, LD and naïve T cell populations based on CD27 and CD45RA expression/lack of expression. In addition, SPICE, a data-mining software application (Exon, Bethesda, MD), was used to analyze large FlowJo data sets from polychromatic flow cytometry and to organize the normalized data graphically. Assay was validated before patient samples were tested and were preformed according to GLP guidelines. Flow cytometry was also performed on rested PBMCs without peptide stimulation to analyze CD4^+^CD25^+^FoxP3^+^ Treg and CD3^−^CD19^+^ B cells in patient PBMC, CD11b^+^CD33^+^HLA-DR^−^ MDSC following the procedures outlined above.

## Statistical Analysis

IFNγ responses (as determined by the ELISPOT assay) at Day 0, 21, 42, and 73 were analyzed by using a general linear model for correlated data.^34^ The mean structure of the model included the main effects and interaction of day and cohort (treated as factors), as well as the effect of stimulation (nested within the day and cohort). A Kronecker-product variance-covariance structure^35^ was used to account for a possible correlation between the unstimulated and stimulated measurements obtained for the same patient at different days. Differences in the IFNγ response between the stimulated and unstimulated cells at Day 21, 42, and 73 were compared between the cohorts by using suitable F-tests with degrees of freedom approximated by the Kenward–Roger method.^36^ The resulting *p* values were adjusted for multiplecomparisons by using a closed testing procedure^37^ at the 5% significance level (two-sided). The model was fitted with the help of SAS v.9.3 software.

## Data and Materials Availability

This study was registered at ClinicalTrials.gov under the identifier NCT01416038.
