# Supplementary material for: Survivin-targeted immunotherapy drives robust polyfunctional T cell generation and differentiation in advanced ovarian cancer patients
Source: Oncoimmunology. 2015 May 7;4(8):e1026529. doi: 10.1080/2162402X.2015.1026529 (PMC4570133; doi:10.1080/2162402X.2015.1026529)
Supplement: KONI_A_1026529_supplemental_material.pdf [file koni-04-08-1026529-s001.pdf]

Supplementary Materials

1. **Methods**

Vaccine injection site histology

In order to identify immune cell infiltration into vaccine injection site, excision-biopsy tissue from subject 11-10 was sectioned and stained with standard H&E and by immunohistochemistry (IHC). For IHC, sections were stained with primary antibodies against human CD3, CD4, CD8 and CD19, followed by biotinylated secondary antibody, ABC reagent and DAB substrate.

Comparison of modified and native peptides in ELISPOT and tetramer assay

To ascertain modified peptides in the vaccine could generate native peptide reactive T cells, both versions of HLA-A1, A2 and A3 peptides were tested in parallel in ELISPOT and in vitro activated CD8 T cell tetramer staining. For ELISPOT assay, PBMC were stimulated overnight with either modified or native peptides and IFN-γ SFU was determined as outlined for Fig 1. For tetramer assay, PBMC were stimulated for 10 days with either native or modified versions of the peptides in parallel and the frequency of tetramer positive CD8 T cells was determined using either tetramer based on modified peptide or using corresponding version of the peptide based tetramer reagent, as outlined for Fig 2.

Flow cytometry for MDSC, Treg and B cells

MDSC were determined in the whole blood by flow cytometry gating strategy that selectively excluded CD3 (T cells), CD19 (B cells), CD14 (mature myeloid cells) and CD56 (NK cells), followed by identifying cells that are $CD11b^{+}CD33^{+}$ and $HLA\text{-}DR^{-}$. Treg cells were

analyzed in PBMC samples by flow cytometry as $CD4^{+}CD25^{+}$ cells that were positive for intracellular expression of FoxP3. B cell percentage in PBMC shown had the phenotype $CD3^{-}$ $CD19^{+}$.

Flow cytometry for polyfunctional CD8-CM T cells

Peptide stimulated and control PBMC were gated for live CD3+CD8+ T cells and the central memory T cells were identified based on CD27 expression and lack of CD45RA expression. Poly-functional CD8-CM T cells, based on their ability to secrete two or more cytokines, were identified with intracellular cytokine staining for IFN-γ, TNF-α and IL2 as outlined for Fig 4.

2. Supplementary Figures:

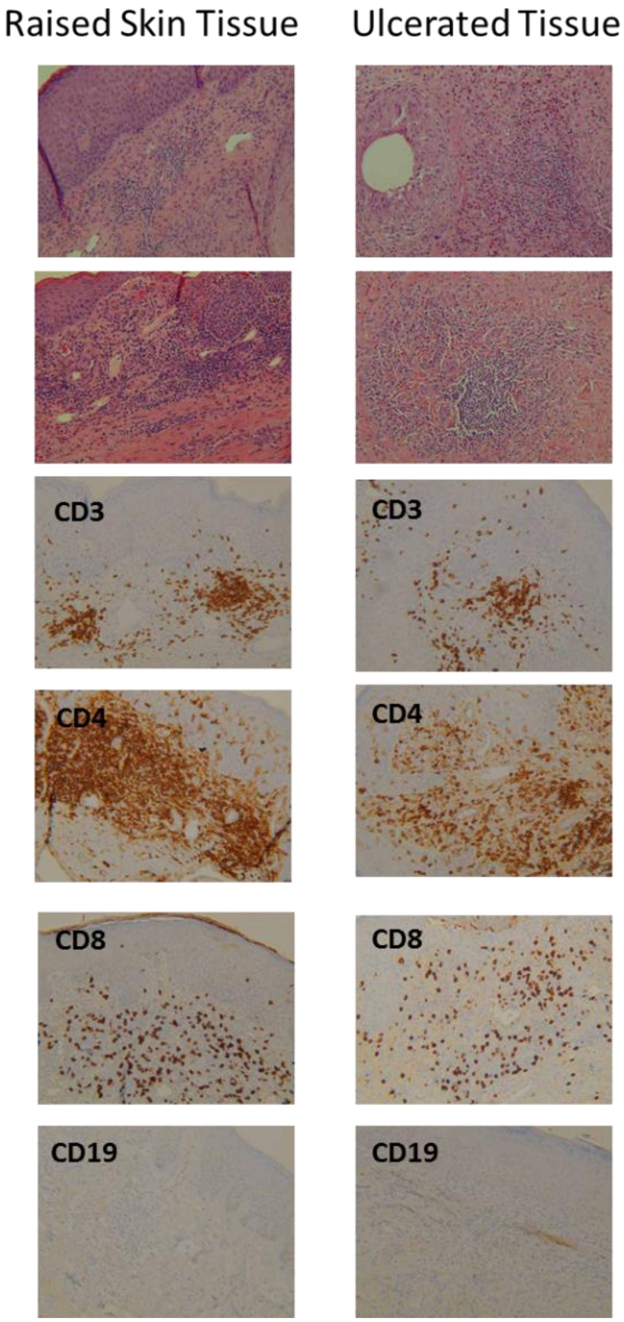

Supplementary Fig. 1: Histology of injection site reaction. Injection site histology was performed on one of the subjects (11-10, cohort C) presenting with lipodystrophy and skin ulceration. The excisional biopsy showed an ulcer associated with needle tract and foreign body granuloma. Based on H&E stain, the epidermis was reported to be unremarkable, but the dermis showed a perivascular lymphocytic infiltrate with eosinophils suggestive of a hypersensitivity reaction. The biopsy was also used in immunohistochemistry for analyzing lymphocyte infiltration, which showed the infiltration of CD4/CD8 T cells but no B cells.

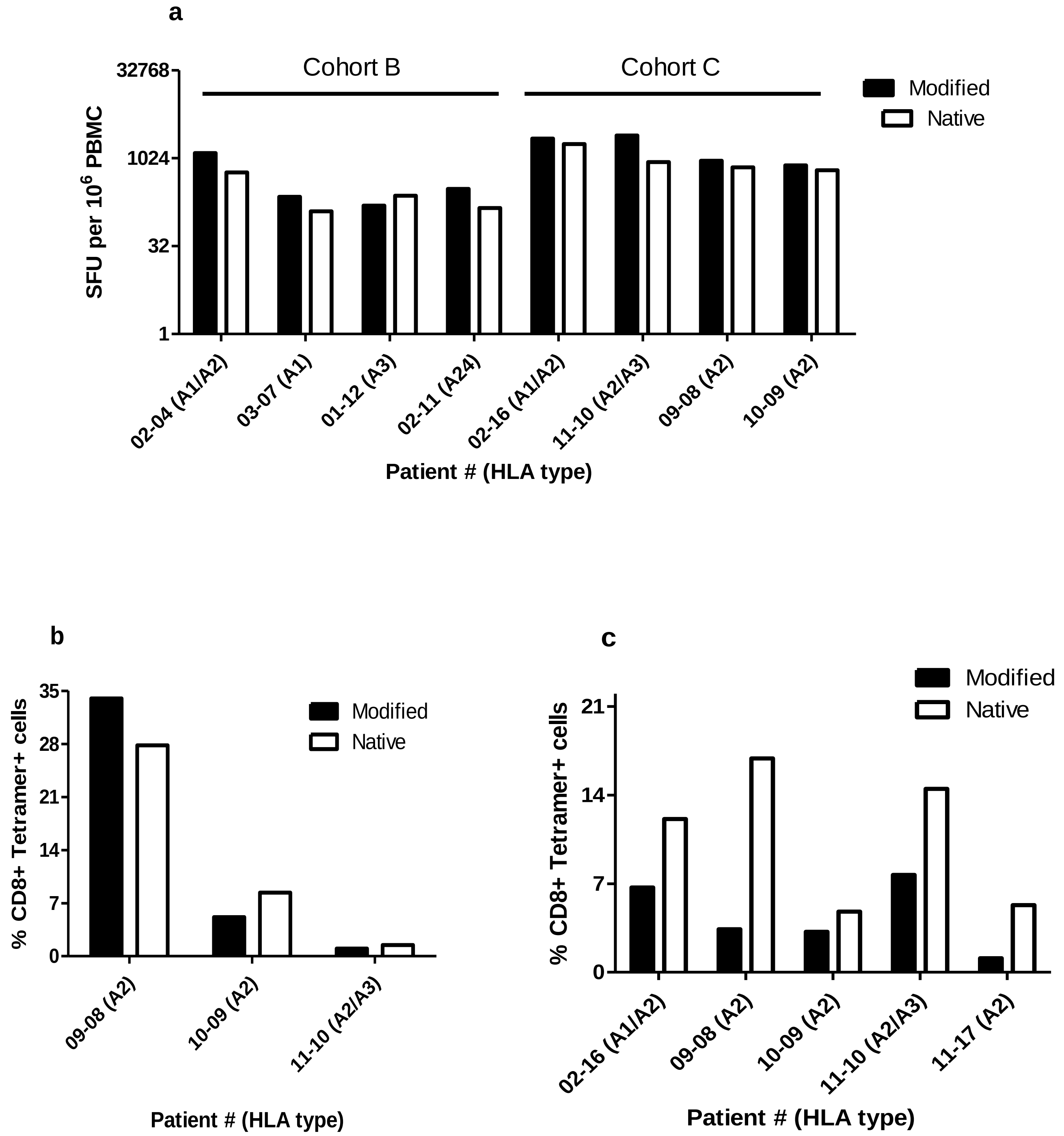

Supplementary Fig. 2: Cross reactivity of patient T cells induced by modified peptides contained in DPX-Survivac to native survivin peptides. In order to ascertain that modified survivin peptides in DPX-Survivac have the ability to generate antigen-specific CD8 T cells that can cross react with corresponding native peptides, ELISPOT (a) and tetramer (b, c) assays were done in parallel using either modified or native HLA-A1, -A2 and -A3 peptides. The results indicate that vaccine-induced T cells react with native peptides as compared to modified peptides in IFN-γ ELISPOT assay (upper panel) and expand tetramer positive CD8 T cells in response to native peptide stimulation (lower panel). b, A2 tetramer used was based on modified peptide and in c, A2 tetramer versions used were corresponding to the peptide version used for stimulation.

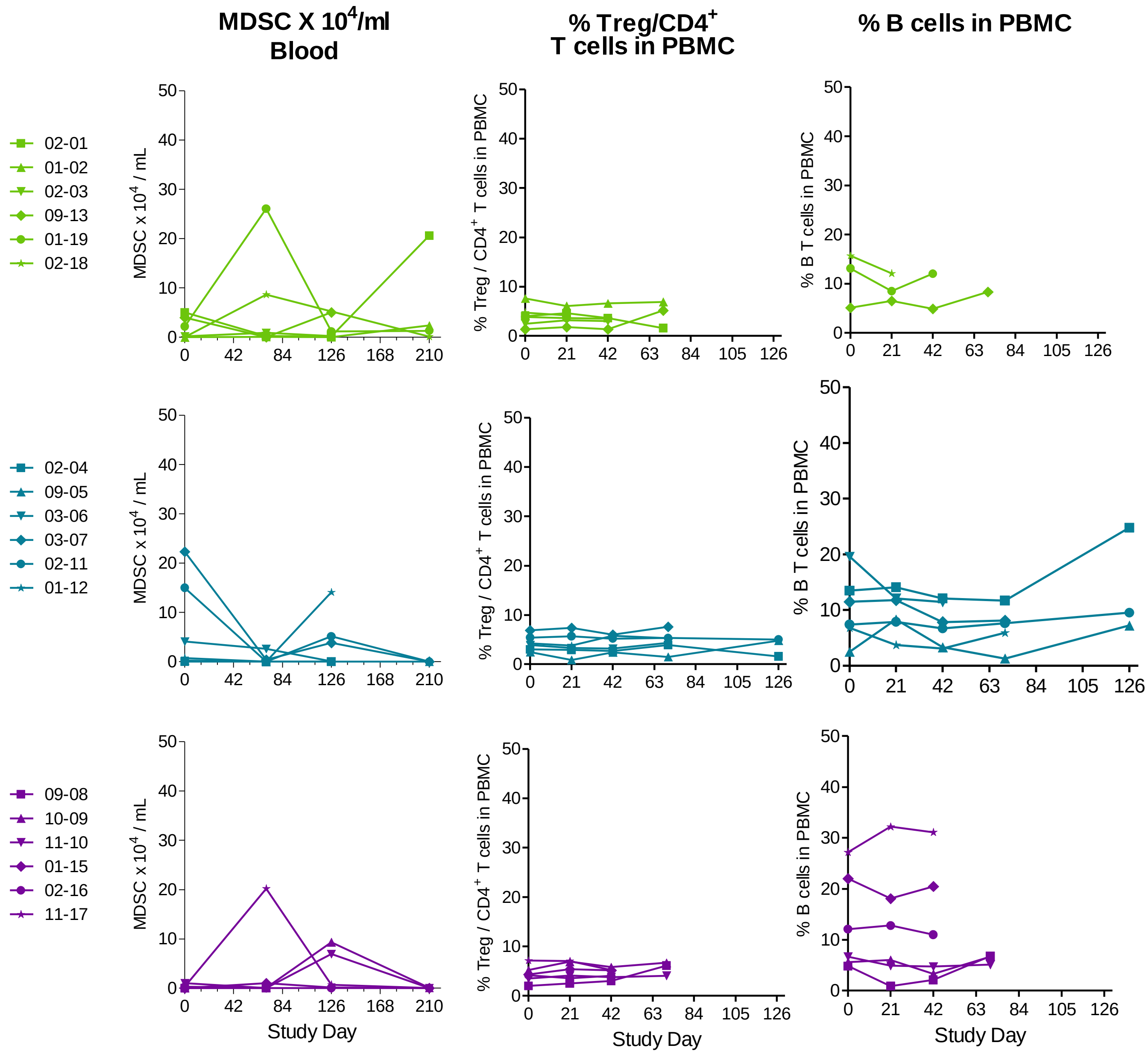

Supplementary Fig. 3: No significant changes in the cell types in peripheral blood that is associated with suppressive immune response. Total MDSC ($CD3^-CD14^-CD19^-CD56^-CD11b^+CD33^+HLA\text{-}DR^-$) in the whole blood, percent Treg/ $CD4^+$ T cells ($CD3^+CD4^+CD25^+FoxP3^+$) and percent B cells ($CD3^-CD19^+$) were analyzed in patient PBMCs by flow cytometry**.** While variability in the frequency of these cell types was observed between subjects and post-vaccination time points, no direct correlation could be made for such observations as vaccine-induced changes.

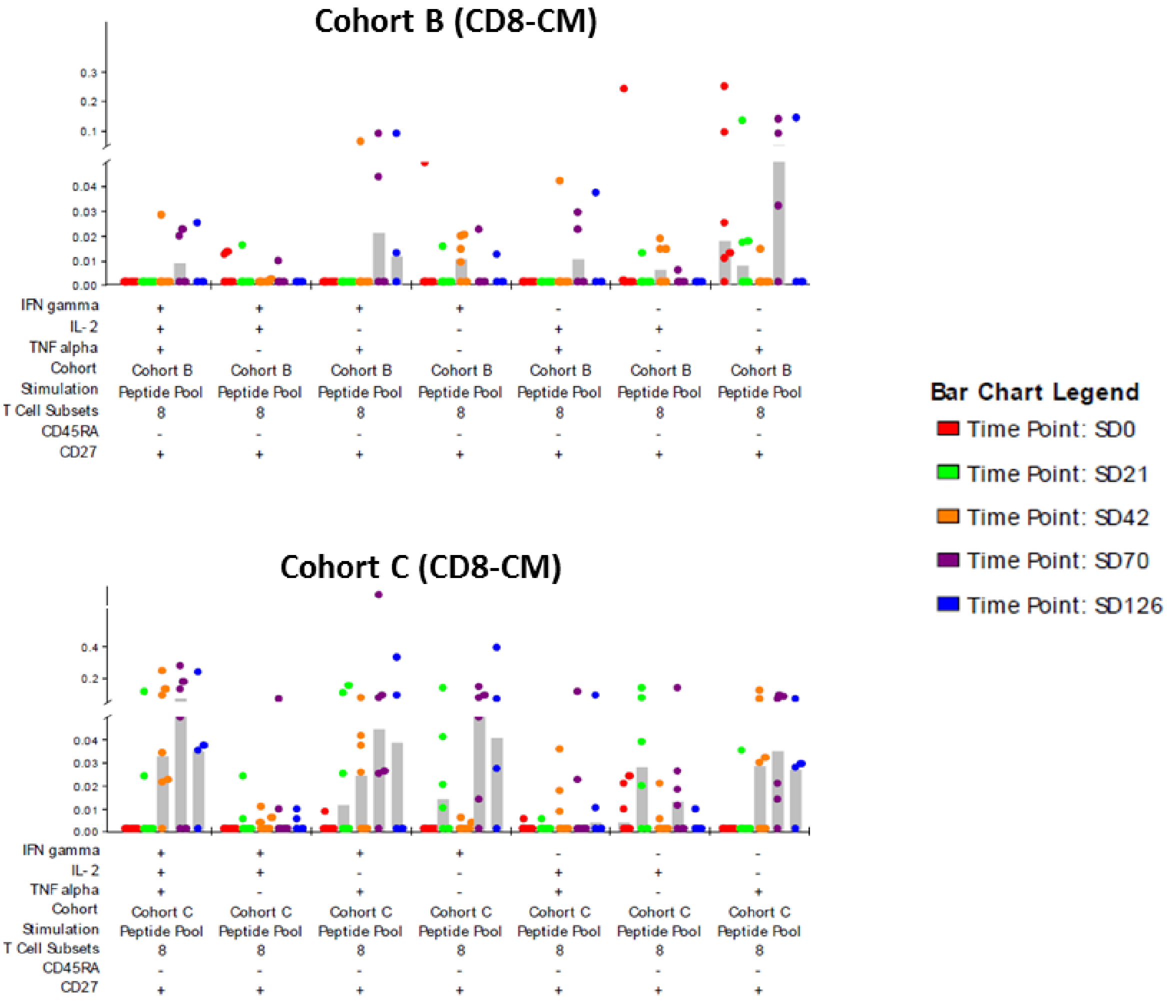

Supplementary Fig. 4: Detailed examination of the generation of polyfunctional CD8$^+$ central memory T cells in cohorts B and C. CD8-CM T cells shown for cohort B and C are shown, representing each data point for individual subject/stimulation at different post vaccination days. The color of the dots represent a given study day and the grey bars represent mean value of multiple/single cytokine positive CD8-CM T cell frequency

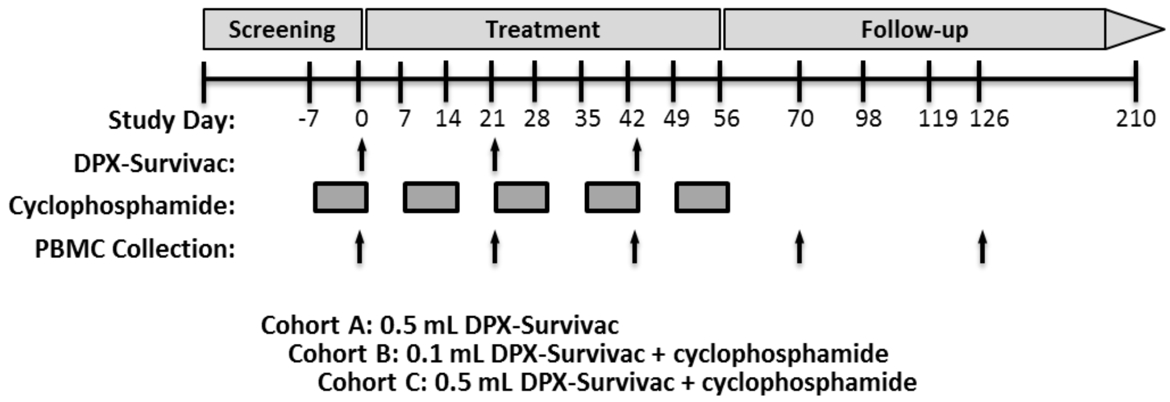

Supplementary Fig. 5: Clinical Trial design. Ovarian cancer patients were recruited into three dose/treatment cohorts as indicated. Cohort A received high dose DPX-Survivac vaccine alone (three doses, three weeks apart) while cohort B and cohort C subjects received oral low dose metronomic cyclophosphamide (50 mg BID) one week on / one week off, along with either low dose or high dose vaccine respectively. Blood for immune monitoring was collected at baseline, during each vaccine dosing and during the follow-up period as indicated. Safety profile and injection site reactions were continuously monitored and recorded.

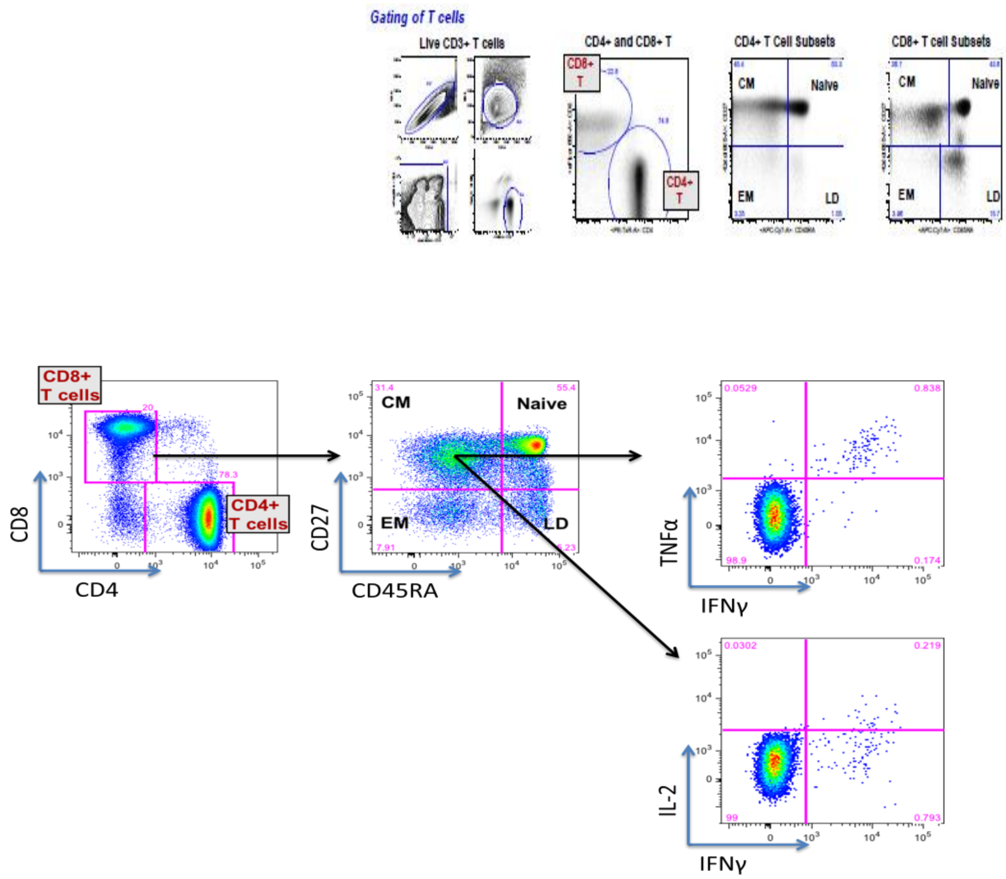

Supplementary Fig. 6: Gating strategy for flow cytometry. Representative data set shown in the lower panel is from subject 11-10 for cytokine analysis on CD8 central memory T cells.

Supplementary Table 1: Safety and reported adverse events which were possibly, probably or definitely related to DPX-Survivac treatment

Toxicity	Total No. of Subjects (19)			Cohort A (0.5 mL)			Cohort B (0.1 mL + CTX)			Cohort C (0.5 mL + CTX)		
	A (7)	B (6)	C (6)	Grade 1	Grade 2	Grade 3	Grade 1	Grade 2	Grade 3	Grade 1	Grade 2	Grade 3
Injection site induration	7	6	6	4	3		5	1		5†	1*	
Injection site erythema	6	6	6	5	1		5	1		4†	1	1*
Injection site pain	5	3	5	4	1		3			5*†		
Injection site edema/ swelling	3	3	2	2	1		3			1	1*	
Injection site pruritus	2	2	3	2			2			2†	1*	
Injection site warmth	1	1	4	1			1			4*†		
Injection site ulceration	1	1	3		1				1		1	2*†
Injection site exfoliation/ dryness	1	-	3	1						3*†		
Injection site extravasation/ discharge	-	-	2							1†	1*	
Injection site rash	-	1	1				1				1*	
Injection site vesicles	1	-	1	1						1*		
Injection site hematoma	2	-	-	2								
Injection site hypersensitivity	1	-	-	1								
Injection site lipodystrophy	-	-	1									1†
Injection site necrosis	-	-	1									1*
Injection site skin infection	-	-	1								1*	
Fatigue	1	4	1	1			4			1		
WBC Count decreased	-	1	-					1				
Chills	1	-	1	1						1*		
Lymphodenopathy	1	-	1	1						1		
Arthalgia	1	-	-	1								
Mobility decreased	1	-	-	1								
Neurophathy peripheral	1	-	-	1								
Muscle spasm	-	1	-				1					
Pyrexia	-	-	1							1		
Myalgia	-	-	1							1		
Influenza-like illness	-	-	1							1†		
Chest discomfort	-	-	1							1*		

*denotes events occurring in same subject; †denotes events occurring in same subject

Supplementary Table 2: Antibody source, catalog number/clone used in flow cytometry assay

Analyte	Fluorochrome	Source	Catalog #
CD3	V450	BD Bioscience	560351
CD8	eFluor 650NC	e-Bioscience	95-0088
CD45RA	APC-Cy7	Biolegend	304128
CD27	Qdot 605	Invitrogen	Q10065
CD4-ICS panel	ECD	Beckman Coulter	6604727
CD4-Treg panel	PerCP Cy5.5	BD Bioscience	560650
IFN-γ	PE-Cy7	BD Bioscience	557643
TNF-α	APC	BD Bioscience	340534
IL-2	Alexa 700	Biolegend	500320
IL-17	PerCP Cy5.5	e-Bioscience	45-7179
Granzyme B	FITC	BD Bioscience	558132
CD25	PE-Cy7	BD Bioscience	557741
FoxP3	APC	e-Bioscience	17-4776-42
CD19	FITC	BD Bioscience	555414
CD11b	APC	BD Bioscience	550019
CD33	PE	BD Bioscience	555450
HLA-DR	PE-Cy7	BD Bioscience	560651
Live/Dead Aqua	Aqua	Invitrogen	L34957
